# Education by Picnic

**Published:** 1889-09-21

**Authors:** 


### "Education by Picnic."

A few days ago an old lady, who was discussing with another the recent strike of dock labourers, expressed profound satisfaction that she was old, and would soon be gone to her rest. She was quite sure that such portentous social changes of all kinds were in progress that very soon the world would be turned completely upside down. What that old lady, herself an experienced teacher of the young, will say if it ever falls to her lot to read a circular just issued by the Edinburgh University authorities, in which they describe their new University Extension scheme as "Education by Picnic," it is impossible to conjecture. Even we ourselves, so broad in our general sympathies as to be practically incapable of measurement, found our eyes dilating so widely when we read it that it almost seemed as if no orbit could contain them. And yet, as Carlyle might say, when you come to think of it—Why not? Edinburgh and the surrounding districts are pre-eminently suited for picnics, and a good deal of education of a very tender kind has been gained by students and others at Melrose and Hawthornden, at Craigmillar and Dalmeny, and at a hundred other romantic spots within reach of the fair city from time immemorial. So that really it is but extending to subjects of education, other than the art of complete love-making, the great and enduring benefits of "progress by picnic." "The popularising of universities proceeds apace," says the circular. Surely we do not need to be told that. "Ten years ago Professor Stuart saw the impossibility of the people coming to the universities. Why, then, should not the universities go to the people?" Most obvious and happy inspiration! If the mountain will not go to Mahomet, why Mahomet had very much better begin to consider whether or not he cannot go the mountain.

The Edinburgh University folk, like those of Oxford, have much faith in the *genius loci*. So have we, and especially when the *locus* is such a place as Edinburgh or Oxford. "Missionary" lecturers are very good sort of people in their way, but they cannot for a moment compare with lecturers who have not only the venerable university itself about them, but the streets, and squares, and terraces of the most beautiful city in the world, the Castle and the Calton Hill, Arthur's Seat and the Pentlands, the Firth of Forth, and the Fife hills, in full vision before them every hour of the day. The mere thought of Edinburgh to him who knows it is like a rush of life from a Swiss mountain. And every young man and woman in Scotland or in England who can afford half-a-guinea in money and a fortnight in time can have a whole course of university lectures by some of the most eminent professors in the world, and a fortnight in Edinburgh to boot. Talk of the canny Scot! Never was there such a bargain offered to reading young men and women in the world before! In addition to the lectures there will be an opening ceremonial and a special service at St. Giles's Cathedral, excursions to Arthur's Seat, the Forth Bridge, the Botanic Garden, and numerous other places; and all personally conducted by specially qualified conductors. Several wealthy citizens, it is said, intend to make the occasion memorable to the visitors by inviting them to social entertainments at their own homes or elsewhere. Clearly the star of demos is in the ascendant! The lectures, it is quite certain, will be the very best of their kind, and when it is considered that they will embrace such subjects as physical science, biological science, history and ethics, political and economic science, literature, art, music, and elocution, it fairly takes the breath away to think of all this being offered freely to all men, together with a fortnight at the university, for ten and sixpence. What would the professors of Carlyle's day have said if they could have peered so far into the future? What would Carlyle himself have said? The times are clearly not only at full gallop, but all riders are beginning to think whether legs ought not to be dispensed with and wings assumed. We had almost forgotten to give the date of the lectures, which would have been an unpardonable omission. But the inauguration ceremony will take place on the 24th of the present month, and will consist of a grand conversazione in the Museum of Science and Art, one of the finest buildings in the world for the purpose. The opening ceremony itself will be worth more than all the money.

